# Review of "Borrelia: Molecular Biology, Host interaction and Pathogenesis", by DS Samuels and JD Radolf

**DOI:** 10.1186/1756-3305-3-52

**Published:** 2010-06-17

**Authors:** Olivier AE Sparagano

**Affiliations:** 1 School of Agriculture, Food and Rural Development, Newcastle University, Newcastle upon Tyne, NE1 7RY, UK

## Review

Writing a book on a single genus is always very risky as it would target only a small number of scientists specialised in such narrow field. However in the case of *Borrelia *it would expand to other field such as vectors and vector-borne diseases, hosts-arthropod interaction, pathogen adaptation to vector and/or mammal host. This book has 18 chapters and it will cover everything you need to know about these Spirochetes from behaviour in the field to sequencing in a molecular laboratory. Each chapter seems to be written by expert in their *Borrelia *field and bring updated information about the state-of-art for research of simply general knowledge for this pathogen.

The most fascinating is to learn how the Spirochetes are adapting to different tick tissues, "re-programming" their genome when moving from a tick vector to a mammalian host and succeeding to by-pass the immune system of both of them to complete their life cycle. Genomic studies are showing the important of the plasmids in *Borrelia *cells while the molecular phylogeny will explain why we have species differences between Europe and the American continent and if it was the host or the tick which introduced the pathogen.

This book would definitely interest researchers and some teachers seeking research-led examples for their lectures on a similar topic but the small number of illustrations would require academic staff finding other visual aids to complement the data presented here. One criticism would be that the editing steps did not prevent a few redundancies between chapters trying to re-explain what Lyme Disease or Relapsing fever are. Nevertheless this book is a fantastic source of information for scientists working on vector-borne diseases and interested in epidemiology, evolution, genomics....

I truly enjoyed reading this book and would recommend it to the readership.

## Competing interests

The author serves on the Editorial Board of the Open Access Journal "Parasites and Vectors".

